# Unveiling the Spectrum of Respiratory Syncytial Virus Disease in Adults: From Community to Hospital

**DOI:** 10.1111/irv.70107

**Published:** 2025-05-16

**Authors:** Koos Korsten, Matthijs R. A. Welkers, Thijs van de Laar, Alex Wagemakers, Peter van Hengel, Peter C. Wever, Eva Kolwijck

**Affiliations:** ^1^ Department of Medical Microbiology and Infection Control Amsterdam UMC Amsterdam the Netherlands; ^2^ Department of Medical Microbiology Onze Lieve Vrouwe Gasthuis Amsterdam the Netherlands; ^3^ Department of Pulmonary Diseases Flevo Hospital Almere the Netherlands; ^4^ Department of Medical Microbiology and Infection Prevention Jeroen Bosch Hospital 's‐Hertogenbosch the Netherlands

**Keywords:** adults, epidemiology, respiratory syncytial virus, RSV

## Abstract

**Background:**

Respiratory syncytial virus can cause severe disease in the older adult population. Three vaccines for RSV are currently market approved but the risk of RSV‐hospitalization in (older) adults from a community level remains elusive. We aimed to estimate the risk of RSV‐hospitalization and characterize the patients that end up in hospital.

**Methods:**

We manually analyzed records of adults aged ≥ 20 with RSV‐infection between 2022 and 2024 in three hospitals in the Netherlands. These hospitals implemented routine RSV‐testing at emergency departments. Using population‐based data in combination with the in‐hospital data, we estimated the population risk of RSV‐hospitalization. Hospital records were analyzed to characterize the role RSV played in their course of disease.

**Results:**

We analyzed 709 RSV cases of whom 503 (70.9%) were hospitalized. Five hundred twenty‐six patients were ≥ 60, and 183 were < 60 years of age. The population RSV‐hospitalization rate was 6–20 per 100.000 patients aged 20–59 years and 43–236 per 100.000 for those ≥ 60. The highest risks were observed in those with COPD (1702 per 100.000) and with congestive heart disease (2383 per 100.000). RSV caused clinically relevant infection in 88% of hospitalized cases but was only mentioned using specific ICD‐codes in 4.4%. Comorbidity was prevalent (88.5%) and exacerbation of underlying disease caused of 46.3% of RSV‐related hospital admissions. ICU admittance was 11.2% and in‐hospital mortality was 8.1%.

**Conclusion:**

The risk of RSV‐hospitalization from the community is low but is increased substantially in those with underlying disease. RSV is often clinically relevant in hospitalized patients by causing exacerbation of underlying disease but is infrequently mentioned in specific ICD‐codes.

## 1 Introduction

Respiratory syncytial virus (RSV) is a leading cause of respiratory infections worldwide [1]. RSV has emerged as a substantial contributor to respiratory morbidity and mortality in the aging population [2]. Previous studies have shown similar or even worse clinical course during hospitalization compared with influenza and COVID‐19 [3, 4, 5]. RSV therapeutics are limited to ribavirin and RSV vaccine development has proved challenging as multiple attempts were unsuccessful and even harmful [1]. The discovery of the neutralization‐specific viral epitopes of the pre‐fusion RSV glycoprotein have boosted the search for effective RSV vaccines [6]. Three vaccines have recently shown to be effective [7, 8, 9] and have been granted U.S. Food and Drug Administration and European Medicines Agency approval [10, 11, 12, 13].

More data is becoming available about the risk of RSV hospitalization from a community perspective [5, 14, 15], which is crucial information to determine the complete burden of RSV disease and calculate cost‐effectiveness of preventive strategies. It has been shown that increasing age enhances the risk of RSV hospitalization [5, 14, 15] as well as underlying disease [16]. However, age and presence of underlying comorbidity are intertwined but are important to untangle to determine the role of each of these factors. There are several limitations in the current data that could impact estimation of the disease burden in (older) adults [17]. Serious outcomes such as the hospitalization rate in the general population are rare as shown by the lack of serious outcomes from large prospective cohort studies [7, 8, 9, 18]. Previous studies that determined incidence rates of RSV‐hospitalization were therefore based on registries and used modelling to determine the population‐based hospitalization rate. These studies relied on ICD‐codes [5, 14, 15] or used clinical case definitions such as ILI [14] to subsequently identify RSV patients within the selected cohort. By design, these studies risk underestimation of the burden of RSV‐related disease by using case‐definitions based on clinical symptoms (e.g., fever in ILI/SARI), that may not be sensitive for RSV [19] and by the lack of routine RSV testing in clinical practice that affects a definitive RSV diagnosis [17]. Also, in patients with comorbidities, RSV might be the trigger for hospitalization but may not be the registered hospital diagnosis (ICD‐code) which could also underestimate the role of RSV. Last, younger adults are not considered at risk for severe RSV disease, which might lead to infrequent RSV testing despite that the presence of underlying disease could put them at risk for developing serious sequela that require hospitalization.

RSV vaccines are entering the market and we need to determine who will benefit most from receiving them. With this study, we aim to provide insight in the proportion of patients in the community that visit an emergency department or require hospitalization due to RSV based on age and comorbidity. Next, characteristics of these patients are determined to evaluate what drives subsequent hospitalization and clinical course to determine who might benefit most from RSV vaccination. Last, we aimed to explore and quantify the degree of underestimation of the true RSV burden from using ICD‐codes or case‐definitions. This can aid interpretation of former studies and help in the design of future studies.

## 2 Methods

### 2.1 Patients and Testing Procedures

RSV testing results were retrospectively collected from adults aged ≥ 20 years that visited the ED of the three participating hospitals in the Netherlands between January 2022 and April 2024. We chose this time period because the epidemiology of RSV was disturbed during the actual COVID‐19 outbreak. We included adults ≥ 20 years to align with population‐based age data. The included hospitals were the Amsterdam University Medical Center (tertiary teaching hospital, Amsterdam), Jeroen Bosch Hospital (large general hospital, 's‐Hertogenbosch), and the Flevo Hospital (general hospital, Almere). All patients that tested positive for RSV were included. Patients with nosocomial infection, defined as a positive test at least 7 days after hospital admission were excluded. To estimate the proportion of patients who had acquired RSV in the community and required hospitalization, we selected two of the participating hospitals based on their defined “catchment area.” This included the Jeroen Bosch Hospital which serves the area of 's‐Hertogenbosch, and the Flevo Hospital which serves the area of Almere. Both hospitals are situated in isolated geographical areas without other hospitals nearby which ensures that patients from those “catchment areas” who needed inpatient care were most likely referred to those hospitals. This analysis was not performed for the Amsterdam UMC because the catchment area was not exclusive because of other hospitals that did not perform universal RSV testing and could therefore not be recruited for participation in this study. All hospitals had implemented routine testing for SARS‐CoV‐2 because the start of the COVID‐19 pandemic in 2020 using the GeneXpert CoV‐2/Flu/RSV test [Cepheid, Sunnyvale, CA]. This resulted in ongoing RSV testing in all patients presenting with respiratory symptoms.

### 2.2 Data Collection

Population‐based demographic data for the predefined catchment areas were collected using the Dutch census data from the StatLine database of Statistics Netherlands (CBS, “Centraal Bureau voor de Statistiek”) [20]. Statistics Netherlands is the principal statistical agency in the Netherlands. We collected regional data on age distribution [21] and national data from 2022 to 2023 on the prevalence of chronic obstructive pulmonary disease (COPD), asthma, diabetes, and congestive heart disease (CHD) [22]. The estimates of the prevalence of comorbidities were extracted from the Netherlands Institute for Health Services Research (NIVEL) data, which is a sentinel surveillance system based on a comprehensive network of approximately 140 general practitioner practices across the Netherlands [22].

To determine the characteristics and assess clinical severity of patients that presented in hospital with RSV infections, we manually analyzed the electronic patient file (EPD) data. We determined general patient characteristics including age, sex, and comorbidities. We scored the presence of the following comorbidities; pulmonary disease (asthma/COPD/interstitial lung disease [ILD]), CHD, metabolic disease (diabetes, renal insufficiency/dialysis), hematologic and solid tumor malignancy, rheumatologic/immunologic/auto‐immune disease, and organ transplantation with active immunosuppression therapy. Secondly, we checked the ED status to extract the reason of presentation, vital signs, infection markers (C‐reactive protein [CRP] and leukocyte count), and radiographic evaluation. In patients admitted to hospital, we subsequently determined the reason for hospitalization, length of stay, culture results, prescription of antibiotics, intensive care unit (ICU) admittance, need for supplementary oxygen or invasive respiratory support, and mortality. In order to determine the presence of an bacterial superinfection, we created a scoring system which involved the presence of radiologic signs of pneumonia (2 points), relevant positive microbiologic culture result (2 points), enhanced infection markers (CRP > 100 mg/L and/or leukocyte count > 11 × 10^9^/L, 1 point), and antibiotic therapy prescribed for presumed pulmonary bacterial superinfection (0.5 points). A bacterial superinfection score below two was classified as “unlikely,” scores ranging from 2 to 3 were classified as “possible” while a score > 3 deemed a bacterial superinfection “likely.” We classified the role that RSV played in course of disease based on the EPD. We defined the categories “primary RSV infection,” “exacerbation of underlying disease caused by RSV,” “bacterial superinfection following RSV,” and “RSV considered irrelevant.” The classification was scored by the authors based on the full hospital records, admission, and discharge letters. In case of an unclear diagnosis about the role of RSV, Table S1 was used to determine the most likely role of RSV and disagreement was solved by consensus between the authors. The clinical relevance of RSV was determined based on both the presence of respiratory symptoms and whether RSV was mentioned by the physicians as a relevant factor in the course of clinical disease. If both factors were present RSV was judged to be clinically relevant, if either or both were absent, it was not. Last, we collected ICD‐10 codes for hospitalized patients to quantify potential underreporting.

### 2.3 Statistical Analysis

The estimation of RSV‐hospitalization incidence was modelled using population‐based data combined with the in‐hospital data. Demographic data from the aforementioned registries were used to obtain the number of people per age group in the predefined study regions (Flevo Hospital Almere and Jeroen Bosch Hospital, 's‐Hertogenbosch). The estimates of the community‐based RSV incidence from previous studies were used (5.4%–7.2% seasonal RSV incidence in older adults ≥ 60; Korsten et al. [18] and 7% total incidence for healthy adults aged 18–60 years; Hall et al. [23]). Combined with the number of people in the population, we extrapolated the number of patients that would theoretically be infected with RSV in the community seasonally. The incidence of RSV admissions from the community (rate per 100,000 persons) was calculated by dividing the number of RSV admissions during the studied period by the population at risk during that same period. Seasons were defined to calculate the incidence of RSV‐hospitalization per RSV season. The seasons were defined retrospectively based on a period of 3 months before and after the peak incidence month of RSV‐hospitalization during that year. We performed the same modeling exercise stratified for patients with COPD, asthma, CHD, and diabetes. Because the most recent data for the prevalence of comorbidities in the general population were from 2022 [22], we only performed these stratified analyses for the season 2022–2023. In order to correct for the confounding influence of age, we provided estimates for these risks per comorbidity stratified per age group (20–39, 40–59, 60–75, and ≥ 75). Next, we characterized the clinical severity of RSV positive in‐hospital patients and compared patients that visited the ED who were, or were not, subsequently admitted to hospital. We did not use imputation to correct for missing data. All analyses were performed in R software, Version 4.0.1 (www.r‐project.org).

Ethical approval for the study was obtained from the Medical Ethics Committee Brabant (Reference Number NW2024‐23), and individual patient informed consent was waived.

## 3 Results

### 3.1 Population Risk of RSV‐Hospitalization

**FIGURE 1:**
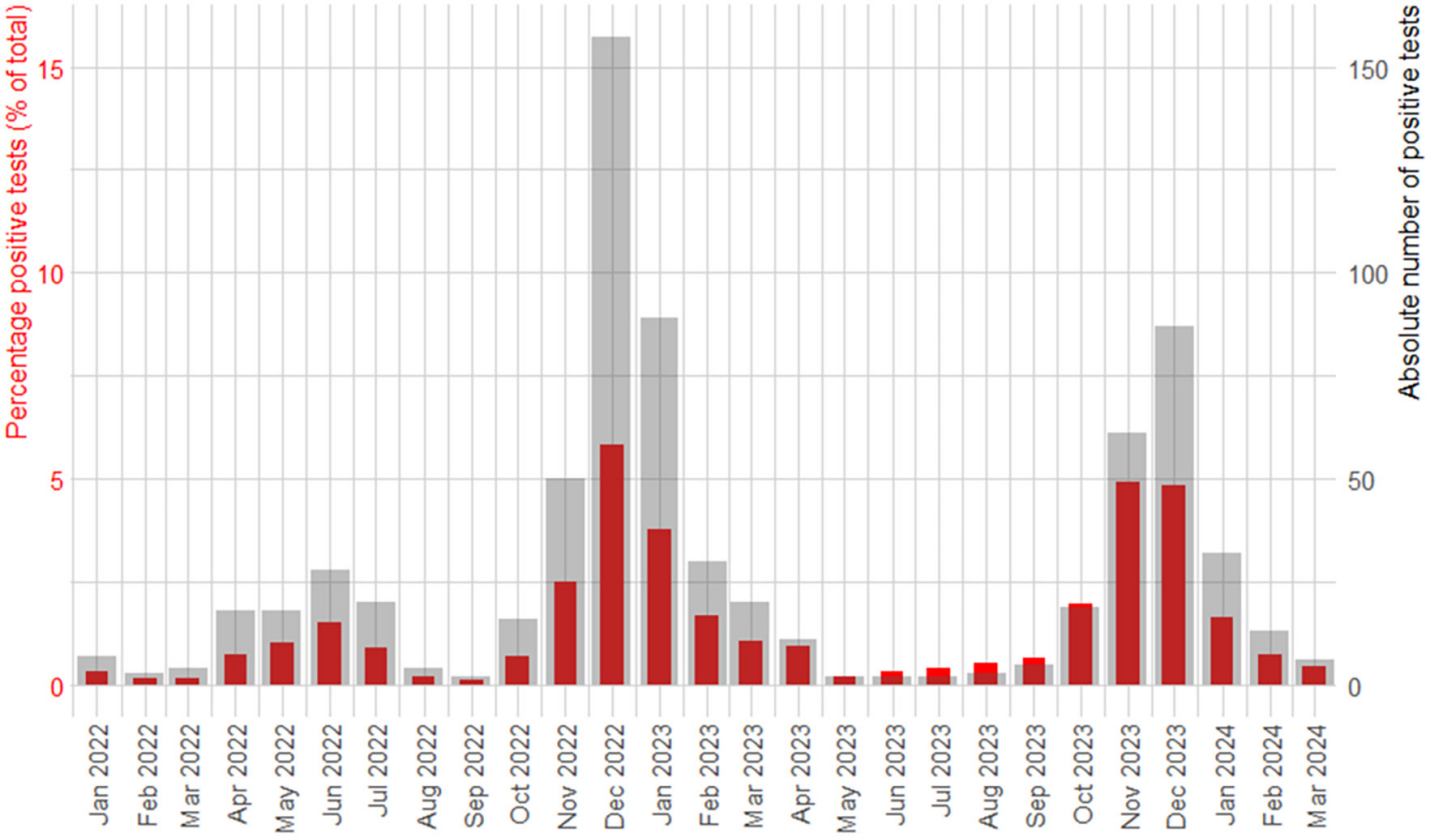
Epidemiology of respiratory syncytial virus (RSV) during the study period for all hospitals combined. The red bars define the percentage of positive RSV tests as a fraction of all performed RSV tests (scale on the left). The grey bars define the absolute number of positive RSV tests (scale on the right).

From January 2022 up to April 2024, we extracted 45,651 individual RSV test results collected from 32,056 inpatients aged ≥ 20 years. After exclusion of nosocomial cases (*n* = 53), 708 individual patients with 709 RSV infections remained, of whom 526 (74%) were aged ≥ 60 years and 183 were between 20–59 years old (Table 1). One 60‐year old patient experienced two RSV infections separated by a 4‐month interval and was therefore included twice. The majority (99.4%) of positive samples were from nasopharyngeal swabs collected in the ED. Two RSV seasons were defined, the 2022–2023 and 2023–2024 season both spanning from September until April based on peak incidence months in December each year. The RSV epidemiology in our study is shown in Figure 1.

**TABLE 1:** Number of respiratory syncytial virus (RSV) cases among total number of individual patients tested.

Site	RSV season	Age 20–59 (RSV/total)	Age 60+ (RSV/total)
Amsterdam University Medical Center	2022–2023	43/2013 (2.1%)	87/2864 (3.3%)
	2023–2024	44/1182 (3.7%)	65/1683 (3.9%)
	Off‐season	21/3915 (0.5%)	36/4916 (0.7%)
Jeroen Bosch Hospital	2022–2023	26/921 (2.8%)	136/2891 (4.7%)
	2023–2024	13/566 (2.3%)	65/1631 (4.0%)
	Off‐season	5/922 (0.5%)	46/2771 (1.7%)
Flevo Hospital	2022–2023	15/582 (2.6%)	57/1032 (5.5%)
	2023–2024	11/534 (2.1%)	25/844 (3.0%)
	Off‐season	5/1058 (0.5%)	9/1731 (0.5%)
Total	2022–2024	183/11,693 (1.6%)	526/20,363 (2.6%)

*Note:* Off‐season is the periods between January 1, 2022, and April 1, 2024, excluding the two defined RSV seasons.

**FIGURE 2:**
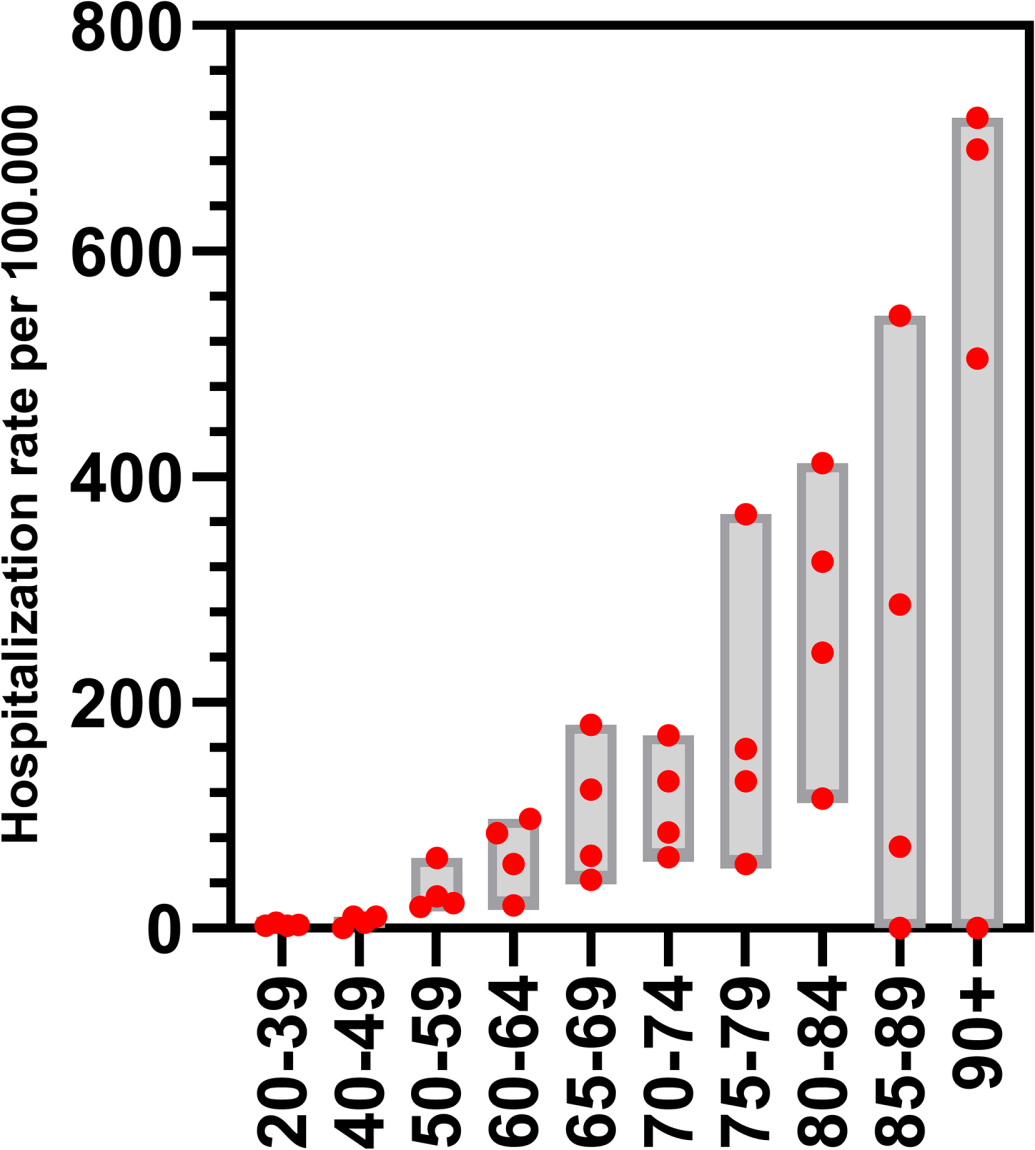
Hospitalization rate from the community setting for various age groups. The bar width represents the lower and upper estimates while the red dots represent the individual estimates per site per season from Table S3.

**FIGURE 3:**
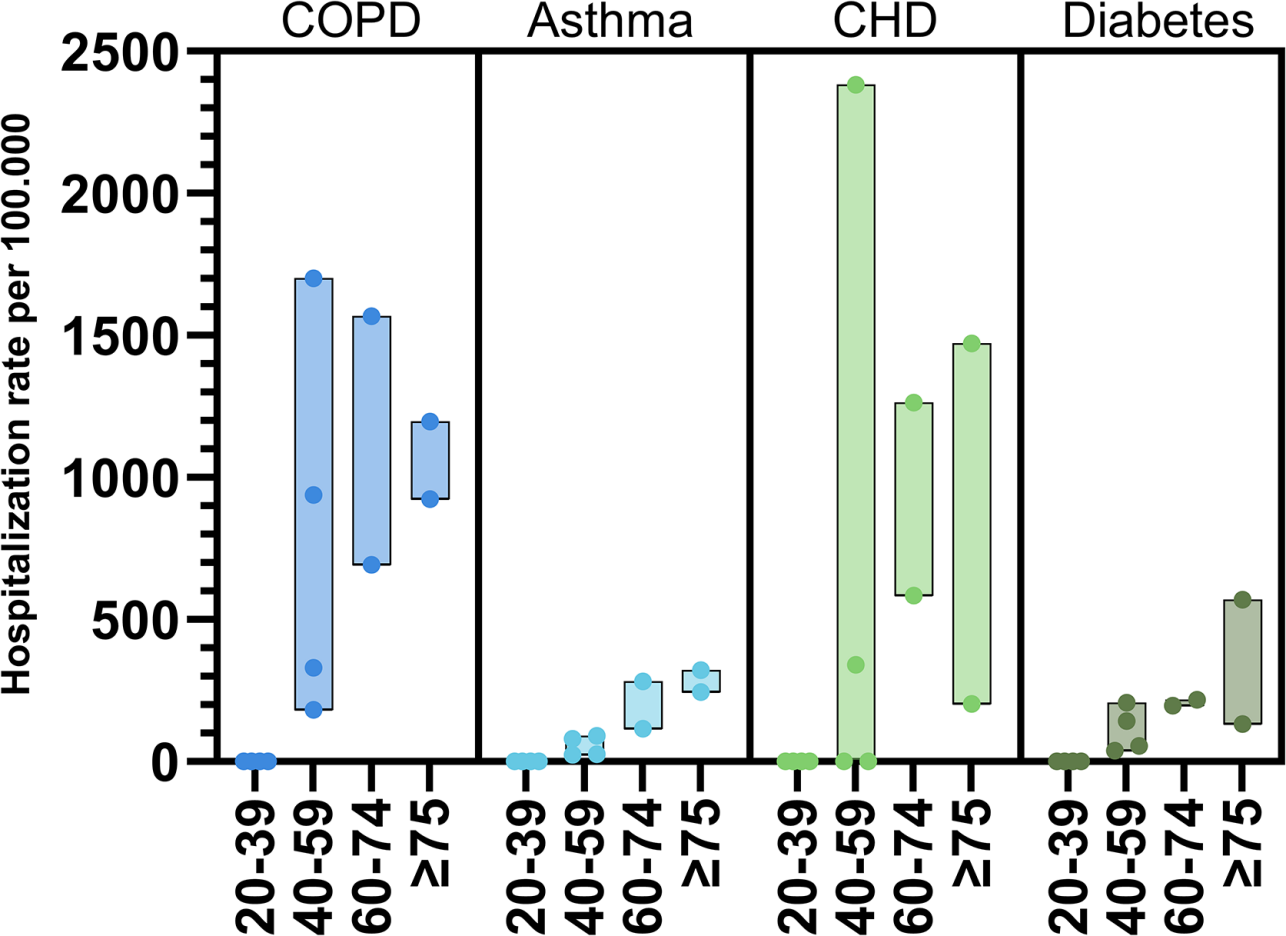
Hospitalization rate from the community setting for various comorbidity groups, stratified by age. The bar width represents the lower and upper estimates while the dots represent the individual estimates per site from Table 3.

In total, 709 RSV episodes were recorded from the ED of whom 503 (70.9%) were subsequently admitted. This resulted in a population risk of RSV‐hospitalization during the RSV season of 6–20 per 100.000 people for patients aged 20–59 years (Table S2) and 43–236 for those aged ≥ 60 years (Table 2). Older age increased the population risk of RSV‐hospitalization up to 718 per 100.000 in those aged ≥ 90 (Figure 2, Table S3). Based on the estimation of the theoretical number of RSV cases in the population, once positive for RSV, the risk of subsequent hospitalization was 81–286 per 100.000 RSV positive patients aged 20–59 years (Table S2) and 598–4372 per 100.000 RSV positive patients in those aged ≥ 60 (Table 2). Subgroup analysis for the hospitalization rate in those with underlying comorbidities showed the highest rates in patients with CHD and COPD (Figure 3 and Table 3).

**TABLE 2:** Respiratory syncytial virus (RSV) epidemiology in adults aged 60 years and older.

		Region Flevo Hospital		Region Jeroen Bosch Hospital	
		Season 2022–2023	Season 2023–2024	Season 2022–2023	Season 2023–2024
Population level	Number of people in the population ≥ 60^a^	42,166	44,107	39,807	40,760
	Estimated RSV incidence (*n*. 5.4%–7.2%)^b^	2277–3036	2382–3176	2150–2866	2201–2935
Emergency department	RSV cases (*n*)	57	25	136	65
	Rate of RSV‐related ED visit (per 100.000 people)^c^	135	57	342	159
	Rate of RSV‐related ED visit (per 100.000 RSV patients)^d^	1877–2503	787–1050	4745–6326	2215–2953
Hospitalized	RSV cases (*n*)	47	19	94	57
	RSV‐hospitalization rate (per 100.000 people)^c^	111	43	236	140
	RSV‐hospitalization rate (per 100.000 RSV patients)^d^	1548–2064	598–798	3280–4372	1942–2590

^a^ Based on regional data [21].

^b^ Estimated based on RSV incidence in adults aged > 60 years (10).

^c^ Number of confirmed RSV cases in this study visiting the ED/hospital divided by the total number of people in the population * 100%, expressed as rate per 100.000 people.

^d^ Number of confirmed RSV cases in this study visiting the ED/hospital divided by the number of estimated RSV cases in the population * 100%, expressed as rate per 100.000 RSV positive cases.

**TABLE 3:** Respiratory syncytial virus (RSV) epidemiology in adults with comorbidity stratified by age.

		Region Flevo Hospital (season 2022–2023)				Region Jeroen Bosch Hospital (season 2022–2023)			
		Age 20–39	Age 40–59	Age 60–74	≥ 75 years	Age 20–39	Age 40–59	Age 60–74	≥ 75 years
Population level	Total population^a^	63,291	60,888	34,172	9935	44,036	41,961	27,715	13,044
	Patients with COPD^b^	190–316	426–2192	2597	1083	132–220	294–1511	2106	1422
	Patients with asthma^b^	5443–7278	5480–6211	3486	934	3787–5064	3776–4280	2827	1226
	Patients with CHD^b^	0–63	61–426	683	984	0–44	42–294	554	1291
	Patients with diabetes^b^	380–949	1400–5297	5092	2275	264–661	965–3651	4130	2987
Emergency department	COPD: rate/100.000^c^ (*n*)	0 (0)	182–938 (4)	693 (18)	923 (10)	0 (0)	331–1702 (5)	1614 (34)	1266 (18)
	Asthma: rate/100.000^c^ (*n*)	14–18 (1)	81–91 (5)	115 (4)	321 (3)	0 (0)	23–26 (1)	283 (8)	326 (4)
	CHD: rate/100.000^c^ (*n*)	0 (0)	0 (0)	732 (5)	203 (2)	0 (0)	340–2383 (1)	1624 (9)	1626 (21)
	Diabetes: rate/100.000^c^ (*n*)	0 (0)	38–143 (2)	275 (14)	132 (3)	0 (0)	55–207 (2)	291 (12)	603 (18)
Hospitalization	COPD: rate/100.000^c^ (*n*)	0 (0)	182–938 (4)	693 (18)	923 (10)	0 (0)	331–1702 (5)	1567 (33)	1196 (17)
	Asthma: rate/100.000^c^ (*n*)	0 (0)	81–91 (5)	115 (4)	321 (3)	0 (0)	23–26 (1)	283 (8)	245 (3)
	CHD: rate/100.000^c^ (*n*)	0 (0)	0 (0)	585 (4)	203 (2)	0 (0)	340–2383 (1)	1263 (7)	1471 (19)
	Diabetes: rate/100.000^c^ (*n*)	0 (0)	38–143 (2)	196 (10)	132 (3)	0 (0)	55–207 (2)	218 (9)	569 (17)

Abbreviations: CHD = congestive heart disease, COPD = chronic obstructive pulmonary disease.

^a^ Based on regional data [21].

^b^ Estimated absolute number of persons with that comorbidity in that region. A range in prevalence is given for the age groups 20–39 and 40–59 based on a spread in estimates within these specific age categories in the source data [22]. Estimates used to calculate these prevalence rates of comorbidity per age group (20–39, 40–59, 60–74, and ≥ 75) can be found in the Supporting Information.

^c^ Number of confirmed RSV cases in this study visiting the ED/hospital with that comorbidity divided by the total number of people in this population * 100%, expressed as rate per 100.000 RSV positive cases (*n* = number of confirmed RSV cases in this study).

### 3.2 In‐Hospital Severity

In total, 709 RSV episodes were examined based on the hospital records. Patient characteristics are shown in Table 4 and stratified per site in Table S4. Underlying comorbidity was present in 83.6%. The most frequent comorbidities were pulmonary (45.3%), and comorbidity was generally more prevalent in the older age group with the exception of rheumatic/immunologic disease, which was more common in the younger age group. Cough was reported in the ED by 85% of patients, while 74.7% of older adults and 55.7% of younger adults reported dyspnea. Fever was reported in 38%, and confirmed at the ED in only 29.9% of cases. Hospitalization was more frequent in older adults as compared with young adults (76.2% versus 55.7%, respectively). There were differences in cohort characteristics per site (Table S4). There was a higher percentage of cases from the tertiary teaching hospital with underlying malignancy and organ transplant and a higher fraction of patients that were < 60 years as compared with the general hospitals. The main difference between the two general hospitals was that patients in the Jeroen Bosch hospital were relatively older (mean age 74 versus 68) and had more frequent CHD (23.0% versus 13.9%) as compared with those from the Flevo Hospital. This age difference aligns with the age distribution based on the demographic data of the areas [21].

**TABLE 4:** Characteristics of the study cohort.

	Age 20–59	Age ≥ 60	Total
	*n* = 183	*n* = 526	*N* = 709
Male	82 (44.8%)	249 (47.3%)	331 (46.7%)
Age (median, [IQR])	47 [35–55]	74 [67–82]	69 [59–79]
Any comorbidity^a^	139 (76.0%)	454 (86.3%)	593 (83.6%)
Pulmonary disease	69 (37.7%)	252 (47.9%)	321 (45.3%)
COPD	21 (11.5%)	178 (33.8%)	199 (28.1%)
Asthma	36 (19.7%)	54 (10.3%)	90 (12.7%)
Congestive heart disease (CHD)	15 (8.2%)	126 (24.0%)	141 (19.9%)
Cardiopulmonary disease^b^	4 (2.2%)	47 (8.9%)	51 (7.2%)
Active malignancy	31 (16.9%)	95 (18.1%)	127 (17.9%)
Hematologic	17 (9.3%)	49 (9.5%)	66 (9.3%)
Solid tumor	14 (7.7%)	47 (8.9%)	61 (8.6%)
Metabolic disease	35 (19.1%)	182 (34.6%)	217 (30.5%)
Diabetes	14 (7.7%)	130 (24.7%)	144 (20.3%)
Chronic renal insufficiency	11 (6.0%)	54 (10.2%)	65 (9.2%)
Hemodialysis	5 (2.7%)	10 (1.9%)	15 (2.1%)
Organ transplant^c^	7 (3.8%)	8 (1.5%)	15 (2.1%)
Rheumatic/immunologic disease^c^	37 (20.2%)	62 (11.8%)	99 (14.0%)
Symptoms (anamnestic)			
Cough	149 (81.4%)	454 (86.3%)	603 (85.0%)
Dyspnea	102 (55.7%)	393 (74.7%)	495 (69.9%)
Fever	83 (45.4%)	187 (35.6%)	270 (38.0%)
Fever measured in the ED (≥ 38°C)	50 (29.6%)	153 (29.9%)	203 (29.9%)
CT value (median, [IQR])	27.5 [22–32]	24.5 [21–29]	24.8 [21–30]
Hospitalization	102 (55.7%)	401 (76.2%)	503 (70.9%)

^a^ From the comorbidities that were noted in Section 2.

^b^ Patients that both have CHD and pulmonary comorbidity.

^c^ With active immunosuppressive therapy or illness.

In 623/709 (88%) episodes, RSV was judged to be a clinically relevant finding in the course of the ED visit or subsequent hospital admission. Clinical severity in episodes in which RSV was clinically relevant is shown in Table 5. Hospitalized patients were older and had higher prevalence of comorbidity (88.5%) of which pulmonary disease was most prevalent (54.8%), followed by CHD (22.4%), diabetes (21.9%), and active malignancy (15.4%). Admittance to an ICU was needed in 11.2% of cases of whom 50% needed invasive respiratory support. In‐hospital mortality was 8.1% in patients in whom RSV was clinically relevant. Stratified for age mortality was highest in those above 75 years of age (13.8%) (Table S5). ICU admission was inversely more frequent in younger adults in which ICU admission was more often deemed feasible. RSV was most often associated with exacerbation of underlying disease in hospitalized patients (46.3%) while in patients that did not require hospital admission, RSV was often judged to be the sole cause of disease (68.8%). Treatment for presumed bacterial superinfection was given in 293 hospitalized cases of whom only 62 (21.2%) had a positive bacterial culture result (Table 5). From the 116 cases suspected for bacterial superinfection following RSV, only 38.8% (45/116) had microbiological proof with a positive bacterial culture result. Based on the bacterial superinfection score 141/293 patients were likely to have an actual bacterial superinfection based on radiographic imaging, culture, inflammatory markers, and prescribed antibiotics (Table S6).

**TABLE 5:** Severity of infection in those with clinically relevant respiratory syncytial virus infection (RSV).

	Emergency department	Hospitalized	Deceased^f^
	*n* = 189	*n* = 434	*N* = 35
Male	91 (48.1%)	192 (44.2%)	17 (48.6%)
Age (median, [IQR])	64 [49–74]	71.5 [63–81]	79 [72–86]
20–39	25 (13.2%)	15 (3.5%)	0 (0%)
40–59	48 (25.4%)	61 (14.1%)	2 (5.7%)
60–74	77 (40.7%)	196 (45.2%)	11 (31.4%)
75 and older	39 (20.6%)	162 (37.3%)	22 (62.9%)
Any comorbidity^a^	152 (80.4%)	384 (88.5%)	30 (85.7%)
Pulmonary disease	72 (38.1%)	238 (54.8%)	19 (54.3%)
COPD	35 (18.5%)	160 (36.9%)	16 (45.7%)
Asthma	29 (15.3%)	58 (13.4%)	2 (5.7%)
Congestive heart disease (CHD)	28 (14.8%)	97 (22.4%)	12 (34.3%)
Cardiopulmonary disease^b^	9 (4.8%)	39 (9.0%)	6 (17.1%)
Active malignancy	46 (24.3%)	67 (15.4%)	5 (14.3%)
Hematologic	24 (12.7%)	37 (8.5%)	3 (8.6%)
Solid tumor	24 (12.7%)	28 (6.5%)	2 (5.7%)
Metabolic disease	46 (24.3%)	139 (32%)	11 (31.4%)
Diabetes	29 (15.3%)	95 (21.9%)	10 (28.6%)
Chronic renal insufficiency	14 (7.4%)	38 (8.8%)	2 (5.7%)
Hemodialysis	6 (3.2%)	7 (1.6%)	0 (0%)
Organ transplant^c^	5 (2.6%)	6 (1.4%)	0 (0%)
Rheumatic/immunologic disease^c^	31 (16.4%)	57 (13.1%)	2 (5.7%)
Severity			
Length of stay for total hospital admission			
Median days [IQR] (all hospitalized patients)	NA	5 [3–8]	6 [2–10]
Median days [IQR] (ICU admitted patients)	NA	10 [8–23]	6 [2–30]
Infiltrate found with chest radiography	NA	164 (37.9%)	17 (48.6%)
Bacterial culture positive	NA	62 (14.3%)	5 (19.2%)
Treated with antibiotics^d^	NA	293 (68%)	26 (74.3%)
Oxygen therapy *n* (%)	NA	331 (76.6%)	33 (94.3%)
Median days [IQR]		3 [2–7]	3 [1–7]
Intensive care unit admission *n* (%)	NA	48 (11.2%)	11 (31.4%)
Median days in the ICU [IQR]		4 [2–11]	6 [2–10]
Invasive ventilation *n* (%)	NA	24 (5.6%)	10 (28.6%)
Median days [IQR]		6 [3–9]	6 [2–8]
Role of RSV			
Primary RSV infection	130 (68.8%)	112 (25.8%)	6 (17.1%)
Factor in exacerbation of underlying disease	50 (26.5%)	201 (46.3%)	17 (48.6%)
Bacterial superinfection after RSV	9 (4.8%)	116 (26.7%)	9 (25.7%)
Indeterminate^e^	0 (0%)	5 (1.2%)	3 (8.6%)

Abbreviations: IQR = interquartile range, NA = not applicable.

^a^ From the comorbidities that were noted in Section 2.

^b^ Patients that both have CHD and pulmonary comorbidity.

^c^ With active immunosuppressive therapy or illness.

^d^ For presumed respiratory bacterial superinfection.

^e^ No definitive role of RSV could be addressed to these cases due to mixed pathologies.

^f^ Deceased patients are also included in the hospitalized group.

### 3.3 ICD Codes

In 429 hospitalized patients with clinically relevant RSV infection, we examined the ICD‐10 codes. RSV specific codes (J20.5 and J21) were only used in 4.4% of these patients. The most frequently used ICD codes were J18 (pneumonia organism unspecified, 84 times, 19.6%) and J40–J47 (chronic lower respiratory diseases, 83 times 19.3%). ICD‐codes that represented disease of the respiratory system (J00–J99) were used for 71.6% of these patients, followed by 9.1% with R00–R99 codes (symptoms of the respiratory or circulatory tract) and 7.9% with I00–I99 codes (diseases of the circulatory system). An overview of these ICD codes can be found in Table S7.

## 4 Discussion

In this study, we investigated the population risk of RSV‐hospitalization and characterized patients that presented in hospital with proven RSV infection. We found a hospitalization rate of 43–236 per 100.000 in adults aged ≥ 60 years (one in 424–2336 people) and observed that the rate of RSV‐hospitalization increased with age, up to 718 per 100.000 in those aged ≥ 90. These estimates are broadly in line with rates from Johannesen and colleagues (38 per 100.000 in 65–74 years up to 588 in those aged ≥ 85) [14] and Havers and colleagues (18.8–90.1 per 100.000 in 60–64 years up to 411.4 in those aged ≥ 75) [15] but higher compared with estimates found by Martinon‐Torres and colleagues (10.8 per 100.000 in 65–74 years up to 55.9 in those aged ≥ 85) [5]. The latter can be explained because RSV cases in their study were selected based on a selective set of RSV‐specific ICD codes for which we show substantial underreporting in our study. The study by Widmer and colleagues in 2014 used a similar approach as used in our study and found a population risk of RSV‐hospitalization of 0.11% for adults aged ≥ 50 years although patients had to consent for participating in the study during their ED visit which could underestimate the incidence [24].

Underlying comorbidities also increased the risk of hospitalization substantially in our study as shown by an upper estimated population risk of RSV‐hospitalization of 1702 per 100.000 in those with COPD (one in 59 patients) and 2383 per 100.000 in patients with CHD (one in 42 patients). This increased risk was also observed in younger adults. The prevalence of comorbidities was high in patients hospitalized with RSV as compared with the population prevalence. This was also true for hospitalized adults aged 20–59 years old in whom 76% had underlying comorbidities. An interesting but diffuse group within the younger adults that warrant further investigation are those who use immunomodulatory medication for rheumatic/immunologic disease which made up 20.2% of the younger adult patients that visited the ED and 25% of hospitalized younger adults. The increased risk of severe disease in the younger adults with comorbidity was also shown in previous literature by Osei‐Yeboah and colleagues [16]. The recent study by Branche and colleagues also estimated incidence based on hospital confirmed cases and found a population risk of RSV‐hospitalization of 0.14%–0.26% (140–260 per 100.000) in adults aged ≥ 65 years [25]. They also identified underlying cardiopulmonary disease as a significant driver of RSV‐hospitalization [25].

In our current study, RSV was judged to be a clinically relevant finding in 88% of in‐hospital visits. Exacerbation of underlying diseases by RSV was found to be the main reason for hospital admission. Of interest, still 25.8% of hospitalizations were judged to be caused by isolated RSV‐infection. ICU admittance rate among patients hospitalized with clinically relevant RSV was 11.2% and in‐hospital mortality was 8.1% although mortality was almost exclusively seen in patients aged ≥ 60 years. Our estimates for in‐hospital severity and mortality, and presence of underlying illnesses are in line with previous literature [3, 26].

The strength of this study is the unbiased RSV testing that was routinely performed since the outbreak of COVID‐19. RSV testing was never routine practice before and case definitions were used to determine who should be tested for RSV. We show that even in the hospital setting, fever is not a valid symptom to trigger respiratory testing for RSV as only 38% of cases reported fever and only 29.9% had confirmed fever in the ED thereby fulfilling the WHO case definition of ILI/SARI [27]. This is in line with our previous study in community‐dwelling older adults [19]. Another strength is the data collection from three different levels of inpatient care ensuring a representative case mix of patients presenting at the hospital with RSV. Local differences in patient characteristics between hospitals were observed which emphasizes the necessity of this multicenter approach. All hospital records were manually analyzed which made it possible to determine the role of RSV in the clinical course of disease. Often RSV plays a role in the exacerbation of underlying disease but is not captured in ICD codes which limits the sensitivity of registry studies [17]. We confirmed this by observing that only 4.4% of hospitalized patients had an RSV specific ICD code, and only 71.6% had a respiratory disease ICD‐code (J00–J99). The degree of underestimation of RSV disease by using certain ICD‐codes is relevant and should be taken into account while interpretating past and future registry studies. Another strength is the inclusion of adults aged 20–59 years old, a group in which the burden of RSV is poorly characterized but in which we show that RSV is relevant in those with underlying disease. The main confounder factor in addressing the risk of severe RSV disease is the relationship between increasing age and presence of comorbidity. By stratifying the risks per age group, we tried to partially correct for this confounding bias. Finally, inclusion of multiple RSV seasons, as performed in this study, is vital because differences in epidemiology vary from year‐to‐year.

This study has also limitations. First, even though we identified 709 inpatients with RSV, numbers were still relatively small hampering risk estimation, especially in subgroup analyses in younger adults and in those with comorbidities. Second, the incidence estimates in the Jeroen Bosch Hospital were higher compared with the Flevo Hospital. Demographic age differences on a population level exist but are unlikely to have caused a difference in the fraction of disease. We cannot exclude local differences in RSV epidemiology, prevalence of comorbidity or spillover of (severe) cases to other hospitals. There were no differences in diagnostic procedures because both hospitals used the same PCR platform and mainly used nasopharyngeal swabs for diagnosing RSV which is the main sample material used in clinical practice. Third, the population prevalence estimates for comorbidity are based on national data collected from a network of general practitioners offices [22]. Although distributed throughout the Netherlands, regional differences may exist. Additionally, prevalence rates of comorbidity differ significantly per age group requiring more detailed stratification in even larger cohorts than used in this study. Fourth, our study period included two clear peaks in RSV incidence but also a smaller, extended summer peak in 2022 (Figure 1). These cases were included in the in‐hospital analyses but were excluded for the evaluation of the incidence of RSV‐hospitalization. We believe this is justified because it is not part of the normal epidemiology of RSV to have a summer peak (in the northern hemisphere). Fifth, the retrospective design of the study can introduce bias in several ways. While universal RSV testing was performed in the included hospitals, we cannot confirm that every patient presenting with respiratory symptoms was tested. Missing RSV cases might underestimate the estimates of the population‐based hospitalization risk. Also, registration of relevant comorbidities can be incomplete or inaccurate. We have tried to obtain the most recent data in relation to the RSV event and address comorbidity status as detailed as possible but were dependent on the quality of previous registration. This can result in underreporting of comorbidities, but also overreporting because we could not always verify whether diagnoses were validly made. This could potentially also impact the precision of the estimates from the stratified analysis of the population‐based hospitalization risk per comorbidity group. The evaluation of the clinical relevance of RSV is also dependent on the written interpretation by the treating physician. By requiring both the presence of clinical respiratory symptoms in combination with a mentioned relevance of RSV in the course of disease by the treating physician, our estimate of the clinical relevance is at most too conservative.

## 5 Conclusion

RSV can cause a substantial burden in hospitalized patients with in‐hospital mortality rates up to 13.8% as shown in our study. New RSV vaccines are currently entering the market and have shown to be effective and can provide protection spanning multiple seasons [7, 8, 9, 28, 29]. Based on the population risk for various age groups combined with the characteristics of those that were hospitalized with RSV in our study, it seems that underlying comorbidity and age, rather than just age should play an important role in decision making who should be eligible to receive vaccination.

## Author Contributions

**Koos Korsten:** conceptualization, investigation, writing – original draft, methodology, writing – review and editing, formal analysis, data curation. **Matthijs R.A. Welkers:** methodology, writing – review and editing, data curation. **Thijs van de Laar:** data curation, writing – review and editing. **Alex Wagemakers:** writing – review and editing, data curation. **Peter van Hengel:** writing – review and editing, data curation. **Peter C. Wever:** conceptualization, writing – review and editing, supervision, methodology. **Eva Kolwijck:** conceptualization, writing – review and editing, methodology, supervision.

## Conflicts of Interest

All authors have completed the ICMJE uniform disclosure form at www.icmje.org/coi_disclosure.pdf and declare: no support from any organization for the submitted work. K.K.; No personal fees received. The Amsterdam UMC has received funding for participation in the following activities; educational activities (expert speaker in Webinar, MedTalks; Webinar, PRIME education; RSV Podcast, Pfizer; and creation of an e‐learning module, Farmacotherapie‐online), participation on an advisory board on the RSV burden in older adults from Pfizer. No other relationships or activities that could appear to have influenced the submitted work.

### Peer Review

The peer review history for this article is available at https://www.webofscience.com/api/gateway/wos/peer‐review/10.1111/irv.70107.

## Supporting information


**Table S1.** Role of RSV.
**Table S2.** Respiratory syncytial virus (RSV) epidemiology in adults aged 20–60 years old.
**Table S3.** Respiratory syncytial virus (RSV) epidemiology stratified by age groups.
**Table S4.** Characteristics of the study cohort stratified by site.
**Table S5.** Severity of infection in those with clinically relevant respiratory syncytial virus infection (RSV) stratified by age group.
**Table S6.** Characteristics of patients treated with antibiotics for presumed bacterial superinfection stratified by bacterial superinfection score.
**Table S7.** ICD codes in patients admitted with clinically relevant RSV infection.

## Data Availability

The data that support the findings of this study are available from the corresponding author upon reasonable request.
