# Supplementary material for: Unveiling the Spectrum of Respiratory Syncytial Virus Disease in Adults: From Community to Hospital
Source: Influenza Other Respir Viruses. 2025 May 16;19(5):e70107. doi: 10.1111/irv.70107 (PMC12081945; doi:10.1111/irv.70107)
Supplement: Supplementary file 1 — Table S1. Role of RSV. Table S2. Respiratory syncytial virus (RSV) epidemiology in adults aged 20–60 years old. Table S3. Respiratory syncytial virus (RSV) epidemiology stratified by age groups. Table S4. Characteristics of the study cohort stratified by site. Table S5. Severity of infection in those with clinically relevant respiratory syncytial virus infection (RSV) stratified by age group. Table S6. Characteristics of patients treated with antibiotics for presumed bacterial superinfection stratified by bacterial superinfection score. Table S7. ICD codes in patients admitted with clinically relevant RSV infection. [file IRV-19-e70107-s001.docx]

SUPPLEMENTARY FILE

Unveiling the spectrum of Respiratory Syncytial Virus disease in Adults: from Community to Hospital

**Content**

**Table S1.** Role of RSV (page 2)

**Table S2**. RSV epidemiology in adults aged 20-60 years old (page 3)

**Table S3.** RSV epidemiology stratified by age groups (page 5)

**Table S4.** Cohort characteristics stratified by site (page 6)

**Table S5.** Severity of infection in those with clinically relevant respiratory syncytial virus infection (RSV) stratified by age group (page 7)

**Table S6.** Characteristics of patients treated with antibiotics for presumed bacterial superinfection stratified by bacterial superinfection score (page 8)

**Table S7.** ICD codes in patients admitted with clinically relevant RSV infection (page 9)

**Table S1.** Role of RSV

| **Role** | **Comorbidity** | **Radiographic**  **imaging** | **Microbiology** | **Antibiotics** | **Clinic** | **Lab** | **Clinical diagnosis** |
| --- | --- | --- | --- | --- | --- | --- | --- |
| **Bacterial superinfection** | All is possible | Lobar  infiltrate | Sputum/BAL/blood culture with bacterial pathogen | Started and continued | Fever, cough | High CRP  (>100 mg/L) | Bacterial pneumonia |
| **Exacerbation respiratory disease** | COPD/Asthma | No lobar infiltrate | No culture / culture negative | Not started, discontinued | Improvement with corticosteroid therapy | CRP <100 mg/L  WBC <11 x10^9^/L | Exacerbation  COPD/Asthma |
| **Exacerbation cardiac disease** | Congestive heart failure | Pulmonary edema | No culture / culture negative | Not started, discontinued | Improvement with diuretics | CRP<100 mg/L  WBC <11 x10^9^/L  NT-proBNP >300 | Decompensatio cordis (acute congestive heart failure) |
| **Primary RSV** | All is possible, immunocompromised | No lobar pneumonia | No culture / culture negative | Not started, discontinued | - | CRP<100 mg/L  WBC <11 x10^9^/L | RSV only |
| **RSV not of importance** | All is possible | - | RSV CT >32 | - | Other diagnosis and no (or little) respiratory symptoms | - | Other diagnosis |

This table is used as a guide to aid in determining the role of RSV when it is not clear from the hospital record which diagnosis is made. CRP = c-reactive protein. WBC = white bloodcell count.

**Table S2**. Respiratory syncytial virus (RSV) epidemiology in adults aged 20-60 years old

|  |  | **Region Flevo hospital** | | **Region Jeroen Bosch hospital** | |
| --- | --- | --- | --- | --- | --- |
|  |  | **2022-2023** | **2023-2024** | **2022-2023** | **2023-2024** |
| **Population level** | Number of people | 121778 | 124179 | 84986 | 85997 |
|  | Estimated RSV incidence (n)^1^ | 8524 | 8693 | 5949 | 6020 |
| **Emergency department** | RSV cases (n) | 15 | 11 | 26 | 13 |
|  | Population-based rate of RSV-related ED visit (per 100.000 people)^3^ | 12 | 9 | 31 | 15 |
|  | Population-based rate of RSV-related ED visit (per 100.000 patients with RSV)^4^ | 176 | 127 | 437 | 216 |
| **Hospitalized** | RSV cases (n) | 14 | 7 | 17 | 8 |
|  | Population-based RSV-hospitalization rate (per 100.000)^3^ | 11 | 6 | 20 | 9 |
|  | Population-based RSV-hospitalization rate (per 100.000 patients with RSV)^4^ | 164 | 81 | 286 | 130 |

^1^ Estimated based on a 7% RSV incidence for healthy adults aged 18-60 years (11) * the susceptible population.

^2^ Number of RSV cases visiting the ED/hospitalized divided by the total number of people in this population *100%, expressed as rate per 100.000 people.

^3^ Number of RSV cases visiting the ED/ hospitalized divided by the number of estimated RSV cases in the population *100%, expressed as rate per 100.000 RSV positive cases.

**Table S3.** Respiratory syncytial virus (RSV) epidemiology stratified by age groups

| Age group | Region Flevo hospital | | | | | | Region Jeroen Bosch hospital | | | | | |
| --- | --- | --- | --- | --- | --- | --- | --- | --- | --- | --- | --- | --- |
|  | 2022-2023 | | | 2023-2024 | | | 2022-2023 | | | 2023-2024 | | |
|  | Population  number^1^ | RSV cases | Hospitalization risk (rate/100.000) | Population  number^1^ | RSV cases | Hospitalization risk (rate/100.000) | Population  number^1^ | RSV cases | Hospitalization risk (rate/100.000) | Population  number^1^ | RSV cases | Hospitalization risk (rate/100.000) |
| 20-39 | 60862 | 2 | 3 | 63291 | 1 | 2 | 42829 | 2 | 5 | 44036 | 1 | 2 |
| 40-49 | 28891 | 3 | 10 | 29232 | 0 | 0 | 19422 | 1 | 5 | 19252 | 2 | 10 |
| 50-59 | 32025 | 9 | 28 | 31656 | 6 | 19 | 22735 | 14 | 62 | 22709 | 5 | 22 |
| 60-64 | 14369 | 12 | 84 | 14688 | 3 | 20 | 10349 | 10 | 97 | 10530 | 6 | 57 |
| 65-69 | 10934 | 7 | 64 | 11552 | 5 | 43 | 8870 | 16 | 180 | 8970 | 11 | 123 |
| 70-74 | 7680 | 10 | 130 | 7932 | 5 | 63 | 8188 | 14 | 171 | 8215 | 7 | 85 |
| 75-79 | 4630 | 6 | 130 | 5222 | 3 | 57 | 5718 | 21 | 367 | 6278 | 10 | 159 |
| 80-84 | 2462 | 6 | 244 | 2614 | 3 | 115 | 3640 | 15 | 412 | 3687 | 12 | 325 |
| 85-89 | 1395 | 1 | 72 | 1403 | 0 | 0 | 2027 | 11 | 543 | 2088 | 6 | 287 |
| ≥90 | 696 | 5 | 718 | 696 | 0 | 0 | 1015 | 7 | 690 | 991 | 5 | 505 |
| Age groups for comparison with the comorbidity data from 2022 (2) | | | | | | | | | | | | |
| 20-39 | 60862 | 2 | 3 | 63291 | 1 | 2 | 42829 | 2 | 5 | 44036 | 1 | 2 |
| 40-59 | 60916 | 12 | 20 | 60888 | 6 | 10 | 42157 | 15 | 36 | 41961 | 7 | 17 |
| 60-75 | 32983 | 31 | 94 | 34172 | 13 | 38 | 27407 | 48 | 175 | 27715 | 25 | 90 |
| 75+ | 4630 | 16 | 346 | 9935 | 6 | 60 | 5718 | 46 | 804 | 13044 | 32 | 245 |

^1^ Based on regional data (1)

**Table S4.** Characteristics of the study cohort stratified by site

|  | **Amsterdam University Medical Center**  **N=296** | **Jeroen Bosch Hospital**  **N=291** | **Flevo Hospital**  **N=122** |
| --- | --- | --- | --- |
| Male | 149 (50.3%) | 130 (44.7%) | 52 (42.6%) |
| Age (median, [IQR])  - 20-39  - 40-59  - 60-74  - 75 and older | 67 [51-75]  - 41 (13.9%)  - 66 (22.4%)  - 122 (41.4%)  - 66 (22.3%) | 74 [64-82]  - 9 (3.1%)  35 (12.0%)  121 (41.6%)  126 (43.3%) | 68 [59-76]  - 6 (5.0%)  - 24 (19.8%)  - 59 (48.4%)  - 33 (27.0%) |
| Any comorbidity^1^ | 253 (85.5%) | 240 (82.5%) | 100 (82.0%) |
| Pulmonary disease  - COPD  - Asthma | 111 (37.5%)  - 55 (18.6%)  - 39 (13.2%) | 142 (48.8%)  - 99 (34.0%)  - 31 (10.7%) | 68 (55.7%)  - 45 (36.9%)  - 20 (16.4%) |
| Congestive heart disease (CHD) | 57 (19.3%) | 67 (23.0%) | 17 (13.9%) |
| Active malignancy  - Hematologic  - Solid tumor | 73 (24.7%)  - 44 (14.9%)  - 30 (10.1%) | 43 (14.8%)  - 17 (5.8%)  - 25 (8.6%) | 11 (9.0%)  - 5 (4.1%)  - 6 (4.9%) |
| Metabolic disease  - Diabetes  - Renal insufficiency (hemodialysis) | 94 (31.8%)  - 54 (18.2%)  - 8 (2.7%) | 84 (28.9 %)  - 62 (21.3%)  - 4 (1.4%) | 39 (32.0%)  - 28 (23.0%)  - 3 (2.5%) |
| Organ transplant^2^ | 10 (3.4%) | 4 (1.4%) | 1 (0.8%) |
| Rheumatic / immunologic disease^2^ | 45 (15.2%) | 36 (12.4%) | 18 (14.8%) |
| Hospitalization | 189 (63.9%) | 214 (73.5%) | 100 (82.0%) |
| Role of RSV  - Primary RSV infection  - Factor in exacerbation of underlying disease  - Bacterial superinfection after RSV  - RSV was not of importance  - Indeterminate^3^ | - 121 (40.9%)  - 93 (31.4%)  - 37 (12.5%)  - 44 (14.9%)  - 1 (0.3%) | - 89 (30.6%)  - 108 (37.1%)  - 61 (21.0%)  - 31 (10.7%)  - 2 (0.6%) | - 32 (26.2%)  - 49 (40.2%)  - 27 (22.2%)  - 12 (9.8%)  - 2 (1.6) |

^1.^ From the comorbidities that were noted in the method section ^2.^ With active immunosuppressive therapy or illness. ^3^ No definitive role of RSV could be addressed to these cases due to mixed pathologies.

**Table S5.** Severity of infection in those with clinically relevant respiratory syncytial virus infection (RSV) stratified by age group

|  | **20-39 years**  **n=15** | **40-59 years**  **n=61** | **60-75 years**  **n=196** | **75+ years**  **n=162** |
| --- | --- | --- | --- | --- |
| Length of stay median days [IQR] | 2 [2-5] | 4 [3-8] | 5 [3-8] | 5 [3-7] |
| Treated with antibiotics^1^ | 9 (60.0%) | 36 (59%) | 133 (68.2%) | 115 (71.9%) |
| Infiltrate found with chest radiography | 6 (40.0%) | 17 (27.9%) | 77 (39.3%) | 64 (39.8%) |
| Bacterial culture positive | 3 (20.0%) | 14 (23.0%) | 32 (16.3%) | 13 (8.0%) |
| Oxygen therapy n(%)  median days [IQR] | 8 (57.1%)  2 [1-2] | 42 (70.0%)  4 [2-8] | 159 (81.1%)  3 [2-6] | 122 (75.3%)  3 [2-7] |
| Intensive care unit admission n(%)  median days in the ICU [IQR] | 3 (21.4%)  46 [24-73] | 11 (18.3%)  11 [5-18] | 26 (13.3%)  4 [2-10] | 8 (5.0%)  3 [2-3] |
| Invasive ventilation n(% of ICU)  median days [IQR] | 2 (66%)  11 [7-16] | 7 (63.6%)  9 [8-11] | 11 (42.3%)  5 [3-9] | 4 (50.0%)  3 [2-4] |
| In-hospital mortality | 0 (0%) | 2 (3.3%) | 11 (5.6%) | 22 (13.8%) |

IQR = interquartile range**. ^1.^** For presumed respiratory bacterial superinfection

**Table S6.** Characteristics of patients treated with antibiotics for presumed bacterial superinfection stratified by bacterial superinfection score

|  | **Unlikely**  **(score <2)**  **n=108** | **Possible**  **(score 2-3)**  **n=44** | **Likely**  **(score >3)**  **N=141** |
| --- | --- | --- | --- |
| Age (median, [IQR])  - 20-39  - 40-59  - 60-74  - 75 and older | 72 [66-82]  - 3 (2.8%)  - 12 (11.1%)  - 47 (43.5%)  - 46 (42.6%) | 69 [60-74]  - 3 (6.8%)  - 7 (15.9%)  - 25 (56.8%)  - 9 (20.5%) | 72 [66-82]  - 3 (2.1%)  - 17 (12.1%)  - 61 (43.3%)  - 60 (42.6%) |
| Any comorbidity^1^ | 100 (92.6%) | 39 (88.6%) | 115 (81.6%) |
| Pulmonary disease  - COPD  - Asthma | 64 (59.3%)  - 41 (38.0%)  - 12 (11.1%) | 22 (50.0%)  - 15 (34.1%)  - 5 (11.4%) | 64 (45.4%)  - 44 (31.2%)  - 17 (12.1%) |
| Congestive heart failure | 25 (23.1%) | 11 (25.0%) | 30 (21.3%) |
| Active malignancy  - Hematologic  - Solid tumor | 17 (15.7%)  - 8 (7.4%)  - 8 (7.4%) | 5 (11.4%)  - 3 (6.8%)  - 2 (4.5%) | 27 (19.1%)  - 16 (11.3%)  - 11 (7.8%) |
| Metabolic disease  - Diabetes  - Renal insufficiency (hemodialysis) | 44 (40.7%)  - 33 (30.6%)  - 3 (2.8%) | 16 (36.4%)  - 14 (31.8%)  - 0 (0.0%) | 35 (24.8%)  - 23 (16.3%)  - 2 (1.4%) |
| Organ transplant^2^ | 1 (0.9%) | 1 (2.3%) | 3 (2.1%) |
| Rheumatic / immunologic disease^2^ | 14 (13.0%) | 5 (11.4%) | 19 (13.5%) |
| **Vital signs and laboratory studies in the ED** | | | |
| Oxygen saturation ED (median [IQR]) | 90 [87-95] | 93 [88-95] | 90 [86- 95] |
| Respiratory rate (bpm, median [IQR]) | 24 [20-28] | 25 [20-28] | 22 [19- 28] |
| Pulse (bpm. median [IQR]%) | 98 [85-110] | 105 [85-121] | 101 [85-115] |
| Temperature (°C, median [IQR])  Fever measured in the ED (≥38°C) | 37.5 [36.8- 38.3]  36 (34.6%) | 37.7 [37.0- 38.6]  19 (43.2%) | 37.8 [36.9-38.5]  65 (47.1%) |
| CRP mg/L (median [IQR]) | 66 [32-112] | 36 [25-56] | 135 [48-236] |
| Leukocyte count x10^9/L (median [IQR]) | 9.6 [6.9-12.6] | 9.1 [7.1-10.4] | 13.3 [9.8-15.9] |
| **Hospitalization severity** | | | |
| Infiltrate found on chest radiography | 0 (0.0%) | 36 (81.8%) | 125 (88.7%) |
| Bacterial culture positive | 0 (0.0%) | 8 (18.2%) | 54 (38.3%) |
| Oxygen therapy | 82 (75.9%) | 34 (77.3%) | 117 (83.0%) |
| Intensive care admission | 12 (11.1%) | 5 (11.4%) | 21 (14.9%) |
| Invasive ventilation | 3 (2.8%) | 3 (6.8%) | 12 (8.6%) |
| Mortality | 9 (8.3%) | 5 (11.4%) | 12 (8.5%) |
| Role of RSV  - Primary RSV infection  - Factor in exacerbation of underlying disease  - Bacterial superinfection after RSV  - Indeterminate^3^ | 49 (45.4%)  55 (50.9%)  3 (2.8%)  1 (0.9%) | 9 (20.5%)  16 (36.4%)  18 (40.9%)  1 (2.3%) | 12 (8.5%)  33 (23.4%)  95 (67.4%)  1 (0.7%) |

IQR = interquartile range ^1.^ From the comorbidities that were noted in the method section ^2.^ With active immunosuppressive therapy or illness. ^3^ No definitive role of RSV could be addressed to these cases due to mixed pathologies.

**Table S7.** ICD codes in patients admitted with clinically relevant RSV infection

| **ICD-code** | **Primary RSV infection**  **N=112** | **Factor in exacerbation of underlying disease**  **N=201** | **Bacterial super-infection after RSV**  **N=116** | **Total**  **N=429** |
| --- | --- | --- | --- | --- |
| J00-J99 Diseases of respiratory system  - J00-06 Acute Upper RTI  - J09-12 Viral pneumonia  - J13-15 Bacterial pneumonia  - J18 Pneumoniae organism unspecified  - J20-22 Other Acute LRTI  - J20.5/21 RSV LRTI*  - J40-47 Chronic lower respiratory diseases  - J80-99 Other respiratory diseases** | 71 (63.4%)  - 1 (0.9%)  - 11 (9.8%)  - 1 (0.9%)  - 22 (19.6%)  - 18 (16.1%)  - 13 (11.6%)  - 1 (0.9%)  - 3 (2.7%) | 149 (74.1%)  - 0 (0%)  - 15 (7.5%)  - 1 (0.5%)  - 10 (5.0%)  - 40 (19.9%)  - 4 (2.0%)  - 77 (38.3%)  - 5 (2.5%) | 87 (75.0%)  - 0 (0%)  - 11 (9.5%)  - 9 (7.8%)  - 52 (44.8%)  - 5 (4.3%)  - 2 (1.7%)  - 5 (4.3%)  - 3 (2.7%) | 307 (71.6%)  - 1 (0.2%)  - 37 (8.6%)  - 11 (2.6%)  - 84 (19.6%)  -63 (14.7%)  -19 (4.4%)  -83 (19.3%)  -11 (2.6%) |
| I00-I99 Diseases of circulatory system  - I20-25 Ischemic heart diseases  - I30-52 Other forms of heart disease  - Other I00-I99*** | 3 (2.7%)  - 0 (0%)  - 2 (1.8%)  - 1 (0.9%) | 28 (13.9%)  - 0 (0%)  - 24 (11.9%)  - 4 (2%) | 3 (2.6%)  - 2 (1.7%)  - 1 (0.9%)  - 0 (0%) | 34 (7.9%)  - 2 (0.5%)  - 27 (6.3%)  - 5 (1.2%) |
| R00-R99 Symptoms, signs and abnormal clinical and laboratory findings, not elsewhere classified  - R00-09 Involving circulatory and respiratory  - R50-69 General symptoms and signs | 21 (18.7%)  - 8 (7.1%)  - 13 (11.6%) | 9 (4.5%)  - 7 (3.5%)  - 2 (1.0%) | 9 (7.7%)  - 5 (4.3%)  - 4 (3.4%) | 39 (9.1%)  - 20 (4.7%)  - 19 (4.4%) |
| A00-B99 Infectious and parasitic diseases  - A30-49 Other bacterial diseases | 0 (0%)  0 (0%) | 0 (0%)  0 (0%) | 3 (2.6%)  3 (2.6%) | 3 (0.7%)  - 3 (0.7%) |
| C00-D48 Neoplasms  - C00-97 Malignant neoplasms | 4 (3.6%)  4 (3.6%) | 0 (0%)  0 (0%) | 1 (0.9%)  1 (0.9%) | 5 (1.2%)  - 5 (1.2%) |
| D50-D89 Diseases of the blood and blood-forming organs and certain disorders involving the immune mechanism | 5 (4.5%) | 2 (1.0%) | 1 (0.9%) | 8 (1.9%) |
| E00-E90 Endocrine, nutritional and metabolic diseases | 0 (0%) | 2 (1.0%) | 0 (0%) | 2 (0.5%) |
| G00-G99 Diseases of the nervous system | 1 (0.9%) | 0 (0%) | 0 (0%) | 1 (0.2%) |
| N00-N99 Diseases of the genitourinary system | 0 (0%) | 1 (0.5%) | 1 (0.9%) | 2 (0.5% |
| S00-T98 Injury, poisoning and certain other consequences of external causes Unknown | 1 (0.9%) | 0 (0%) | 1 (0.9%) | 2 (0.5%) |
| Z00-99 Factors influencing health status and contact with health services | 0 (0%) | 0 (0%) | 1 (0.9%) | 1 (0.2%) |
| ICD-code unknown | 15 (13.4%) | 8 (4.0%) | 10 (8.6%) | 33 (7.7%) |

* Specific code for acute bronchitis/bronchiolitis due to respiratory syncytial virus (also included in J20-22 group) ** Combination of ICD codes for other respiratory diseases of the interstitium (J80-84), suppurative and necrotic conditions (J85-86), pleural disease (J90-94), other respiratory diseases (J95-99) and COVID infection (U07.1) *** Combination of ICD codes for other circulatory diseases including pulmonary heart disease and circulation (I26-28), Cerebrovascular diseases (I60-69) and diseases of arteries, arterioles and capillaries (I70-79).

REFERENCES

1. Inwoners per gemeente [Internet]. 2024 [cited 9th July 2024]. Available from: <https://www.cbs.nl/nl-nl/visualisaties/dashboard-bevolking/regionaal/inwoners>.

2. Personen naar bij de huisarts bekende diagnose; leeftijd, geslacht [Internet]. 2022 [cited 9th July 2024]. Available from: <https://opendata.cbs.nl/#/CBS/nl/dataset/83110NED/table?dl=A9370>.
